# PTPRD-inactivation-induced CXCL8 promotes angiogenesis and metastasis in gastric cancer and is inhibited by metformin

**DOI:** 10.1186/s13046-019-1469-4

**Published:** 2019-12-05

**Authors:** Won Jung Bae, Ji Mi Ahn, Hye Eun Byeon, Seokwhi Kim, Dakeun Lee

**Affiliations:** 1 Department of Pathology, Ajou University School of Medicine, 164, Worldcup-ro, Yeongtong-gu, Suwon-si, Gyeonggi-do 16499 Republic of Korea; 2 Institute of Medical Science, Ajou University School of Medicine, Suwon, Republic of Korea

**Keywords:** Angiogenesis, CXCL8, Gastric cancer, Metastasis, PTPRD

## Abstract

**Background:**

Protein tyrosine phosphatase receptor delta (PTPRD) is frequently inactivated in various types of cancers. Here, we explored the underlying mechanism of PTPRD-loss-induced cancer metastasis and investigated an efficient treatment option for PTPRD-inactivated gastric cancers (GCs).

**Methods:**

PTPRD expression was evaluated by immunohistochemistry. Microarray analysis was used to identify differentially expressed genes in PTPRD-inactivated cancer cells. Quantitative reverse transcription (qRT-PCR), western blotting, and/or enzyme-linked immunosorbent assays were used to investigate the PTPRD-CXCL8 axis and the expression of other related genes. An in vitro tube formation assay was performed using HUVECs. The efficacy of metformin was assessed by MTS assay.

**Results:**

PTPRD was frequently downregulated in GCs and the loss of PTPRD expression was associated with advanced stage, worse overall survival, and a higher risk of distant metastasis. Microarray analysis revealed a significant increase in CXCL8 expression upon loss of PTPRD. This was validated in various GC cell lines using transient and stable PTPRD knockdown. PTPRD-loss-induced angiogenesis was mediated by CXCL8, and the increase in CXCL8 expression was mediated by both ERK and STAT3 signaling. Thus, specific inhibitors targeting ERK or STAT3 abrogated the corresponding signaling nodes and inhibited PTPRD-loss-induced angiogenesis. Additionally, metformin was found to efficiently inhibit PTPRD-loss-induced angiogenesis, decrease cell viability in PTPRD-inactivated cancers, and reverse the decrease in PTPRD expression.

**Conclusions:**

Thus, the PTPRD-CXCL8 axis may serve as a potential therapeutic target, particularly for the suppression of metastasis in PTPRD-inactivated GCs. Hence, we propose that the therapeutic efficacy of metformin in PTPRD-inactivated cancers should be further investigated.

## Background

Gastric cancer (GC) is the fifth most common cancer and the third leading cause of cancer-related deaths worldwide [1, 2]. With improvements in diagnostic surveillance, advanced surgical techniques, and improved chemotherapeutic and targeted agents, the survival of patients with GC has been extended. However, approximately half of all patients diagnosed with advanced GC (AGC) still die from recurrent disease or distant metastasis [3, 4]. Although recent high-throughput genomic studies have identified many oncogenic mutations in GC—suggesting new molecular classifications based mainly on gene expression profiles [5, 6]—the underlying mechanism of GC metastasis remains elusive. Thus, studies investigating the mechanism of cancer metastasis and the corresponding therapeutic strategies are necessary.

Signaling pathways are regulated by maintaining a balance between activators, such as protein tyrosine kinases and repressors, such as protein tyrosine phosphatases (PTPs) [7]. To date, studies on cancer biology and drug development have primarily focused on tyrosine kinase inhibitors (TKIs) and thus, there is relatively little understanding regarding the contribution of PTPs to cancer. PTPs have been shown to be inactivated in a number of human cancers, implying that PTPs may have tumor suppressive roles [8]. The PTP receptor-type D (*PTPRD*) gene is frequently inactivated by genetic (mutation, deletion, or copy number loss) or epigenetic (methylation) modifications in various human cancers, including head and neck squamous cell carcinoma (HNSCC) [9], lung cancer [10], cutaneous squamous cell carcinoma [11], glioblastoma [12], and malignant melanoma [13].

It has previously been shown that phosphorylated STAT3 (p-STAT3) is a substrate of PTPRD and that cancer-specific mutations in *PTPRD* abrogate the ability of the phosphatase to dephosphorylate STAT3 [12]. PTPRD is also required for appropriate cell-to-cell adhesion, through its interaction with E-cadherin and β-catenin/T-cell factor signaling [14]. Therefore, exogenous expression of PTPRD inhibits cell growth in human glioblastoma [12], suppresses colon cancer cell migration [14], and decreases cell viability by inducing apoptosis in melanoma cells [13], indicating that the loss of PTPRD promotes an aggressive cancer phenotype. However, the role of PTPRD is still not well understood in the context of GC.

Meanwhile, epidemiological studies have shown that hyperglycemia increases the prevalence and mortality rate of certain malignancies. Experimental studies have supported this finding by demonstrating that hyperglycemia can promote the proliferation and invasion of cancer cells, induce apoptotic resistance, and enhance the chemoresistance of cancer cells [15, 16]. In line with this, the metabolic reprogramming of cancer cells induced by antidiabetics results in a significant decrease in the risk of mammary cancer in animal models [17]. The potential effect of metformin on cancer risk has also been suggested in humans [18]. Although recent studies have identified the underlying mechanisms whereby metformin inhibits cancer progression [19, 20], the anticancer effect of metformin is not yet well established.

Therefore, in this study, we aimed to investigate the role of PTPRD in GC, with a focus on its role in cancer metastasis. In addition, we aimed to identify an effective treatment strategy for PTPRD-inactivated GC. Based on our results, we conclude that metformin may be an attractive treatment option for PTPRD-inactivated cancers.

## Materials and methods

### Patients and tissue samples

We collected paraffin-embedded tissues from patients with GC who underwent gastrectomy between January 2005 and December 2006 at the Ajou University Hospital and whose tumors were pathologically diagnosed as T1b (submucosal invasion) or higher. Clinical data were retrieved from patient medical records. Patients were excluded if they had been treated with pre-operative chemotherapy or radiotherapy. Patients who had distant metastasis at the time of surgery were also excluded. Finally, a total of 332 patients were selected for further analysis. The median follow-up duration of patients in the study was 72.4 months. Pathological stages were determined based on the American Joint Committee on Cancer (AJCC), 7th edition. Overall survival (OS) time was measured from the date of surgery to the date of death or the last follow-up visit. Disease-free survival (DFS) time was defined as the interval between the date of surgery and the first recurrence or death. This study was performed in accordance with the code of ethics of the World Medical Association (Declaration of Helsinki) and was approved by the Institutional Review Board of the Ajou University Hospital (AJIRB-MBR-KSP-18-510).

### Immunohistochemistry and in situ hybridization

Tissue microarray (TMA) analysis was performed on formalin-fixed paraffin-embedded tissues by punching two 2 mm cores in each block. Immunohistochemical staining was performed on 4 μm-thick sections of formalin-fixed paraffin-embedded tissues with an anti-PTPRD antibody (LSBio, Seattle, WA, USA; 1:100), using a BenchMark XT® automated immunostainer (Ventana Medical Systems, Tucson, AZ, USA). Tumors showing strong cytoplasmic staining for PTPRD in more than 50% of the tumor area and normal gastric mucosa were classified as PTPRD-high, whereas those with moderate staining intensity were classified as PTPRD-intermediate, and those with low or no PTPRD staining were classified as PTPRD-low. All immunohistochemistry (IHC) slides were analyzed independently by two experienced pathologists (SK and DL). Epstein-Barr virus (EBV) RNA was detected by an automated EBER staining method, using the Ventana Benchmark in situ hybridization system (Ventana Medical Systems), according to the manufacturer’s protocol.

### Cell lines

The human GC cell lines, MKN45, MKN74, SNU1, SNU216, SNU668, AGS, and NCI-N87 were purchased from the Korean Cell Line Bank (KCLB) and were maintained at 37 °C in a humidified atmosphere containing 5% CO_2_, in RPMI 1640 (Hyclone, South Lagan, UT, USA) supplemented with 10% fetal bovine serum (FBS) and 1% penicillin and streptomycin. Human umbilical vein endothelial cells (HUVECs) were purchased from the American Type Culture Collection (ATCC) and were cultured in endothelial cell medium (ECM; ScienCell, Carlsbad, CA, USA) at 37 °C in a humidified atmosphere containing 5% CO_2._ All cell lines were routinely tested for mycoplasma contamination.

### Reagents and antibodies

The following regents and antibodies were used: U0126 (Enzo Life Sciences, Farmingdale, NY, USA; 20 μM); S3I-201 (AdooQ, Irvine, CA, USA; 100 μM); metformin (1,1-dimethylbiguanide hydrochloride; Sigma-Aldrich, St Louis, MO, USA; 1–5 mM); PTPRD (1:500; Aviva Systems Biology, San Diego, CA, USA); and antibodies against phospho-(p) and total ERK and STAT3 (tyrosine 705 and serine 727), E-cadherin, N-cadherin, vimentin, snail, slug (1:500–1000; Cell Signaling Technology, Danvers, MA, USA), and GAPDH (1:10,000; Novus Biologicals, Littleton, CO, USA).

### Lentiviral transduction

*PTPRD* shRNA (bacterial glycerol stocks of five different sequences) and the non-targeting scrambled shRNA, pLKO.1-puro, were purchased from Sigma Aldrich. Each DNA sample (2 μg) was co-treated with psPAX2 (Sigma Aldrich, 1.5 μg), pMD2.G (Sigma Aldrich, 0.5 μg), and E-fection (2 μg/μg DNA) for lentiviral production in 293TN cells. Viruses were harvested at 24 and 48 h. Lentiviral constructs and 8 μg/mL polybrene (Chemicon, Billerica, MA, USA) were used to transfect MKN74 cells, which were then selected with puromycin (1 μg/mL), to generate stable cell lines. The efficiency of *PTPRD* mRNA knockdown by the five different shRNA sequences was tested by qRT-PCR and shPTPRD #3 was chosen for subsequent experiments.

### DNA transfection

SNU216 and AGS cells were incubated in six-well plates (7 × 10^5^ cells/well) for 24 h and were then transfected with shNS or shPTPRD (SIGM) using Lipofectamine 3000 (Invitrogen, Carlsbad, CA, USA), according to the manufacturer’s instructions.

### siRNA transfection

Cells were incubated in six-well plates (5–7 × 10^5^ cells/well) for 24 h and then transfected with a negative control, *CXCL8*, or *PTPRD* siRNA (Bioneer, Daejeon, Korea) using Lipofectamine 3000, according to the manufacturer’s instructions.

### Western blotting

Cells were lysed in PRO-PREP protein extraction solution (Intron, Seoul, Korea). Proteins were resolved by sodium dodecyl sulfate-polyacrylamide gel electrophoresis, transferred to PVDF membranes, and then probed with specific antibodies. Protein levels were detected using horseradish peroxidase-conjugated secondary antibodies and enhanced chemiluminescence reagents, as per the manufacturer’s instructions (Amersham Biosciences, Boston, MA, USA).

### Cell proliferation assay

Cell proliferation was evaluated using the EZ-Cytox cell viability assay kit (Daeil Lab Service, Seoul, Korea). Briefly, 5 × 10^4^ cells were seeded in 12-well plates. Cells were cultured for 1, 3, or 5 days and then treated with culture medium (300 μL) and EZ-Cytox solution (30 μL) and incubated for 2 h at 37 °C. Absorbance was measured at 450 nm using a Synergy H1 hybrid multi-mode microplate reader (BioTeK, Winooski, VT, USA).

### Transwell migration/invasion assay

Transwells containing 8-μm pores (Corning, Lowell, MA, USA) and Matrigel (BD Biosciences, San Diego, CA, USA) were used for invasion assays. shNS- or shPTPRD-transfected MKN74 and SNU216 cells were seeded into the upper transwell chamber in culture medium at 2.5 × 10^5^ cells/well for migration or 5 × 10^5^ cells/well for invasion assays, and were incubated for 2 days. Cells were fixed in 100% methanol and were stained with hematoxylin/eosin. Cells remaining on the upper surface of the filter were removed with a cotton swab. The filter was then mounted onto a cover slip and images were taken at 100× magnification. Cells were counted in five different fields of each well using Image J software.

### RT−/qRT-PCR

Total RNA was extracted from cells using TRIzol reagent (Invitrogen), according to the manufacturer’s instructions. RNA was reverse transcribed using Accu-Power RT PreMix (Bioneer). The resulting cDNA samples (1 μg/mL) were then amplified using AccuPower PCR PreMix (Bioneer). RT-PCR products were resolved on a 2% agarose gel and stained with SYBR® Safe DNA gel stain (Invitrogen). qPCR was performed on cDNA samples using SYBR Premix Ex Taq (Takara Bio, Shiga, Japan) on a Thermal Cycler Dice Real Time System III (Takara). The relative mRNA levels of target genes were normalized to β-actin mRNA levels, and analyzed using the comparative Ct method (ΔΔCt). The sequences of the specific primers used in this study are detailed in Additional file 1.

### ELISA

Media were harvested at the indicated times and stored at − 20 °C until analysis. The concentration of IL-8 in the culture media was determined by ELISA (R&D Systems, Abingdon, UK), according to the manufacturer’s instructions. Absorbance was measured at 450 nm using a Synergy H1 hybrid multi-mode microplate reader.

### Tube formation assay

Fifty microliters of in vitro angiogenesis ECM gel (Millipore, Burlington, MA, USA) was added to cold 96-well culture plates and allowed to solidify at 37 °C for 1 h. HUVECs (2 × 10^4^ cells/well) were seeded onto the ECM gel and cultured, as described above, at 37 °C in a humidified atmosphere containing 5% CO_2_ for 6–10 h. The formation of polygonal tubes was assessed at 100× magnification using an Olympus microscope (Olympus, Tokyo, Japan).

### Methylation-specific PCR

Genomic DNA was extracted from peripheral blood leukocytes using a genomic DNA extraction kit (Qiagen, Valencia, CA, USA), according to the manufacturer’s instructions. Bisulfite modification of genomic DNA was performed using an EZ DNA methylation kit (ZymoResearch, Irvine, CA, USA). Primer sequences used for PTPRD methylation-specific PCR were as follows: methylation F: 5′-AGG AGT CGG GAG TCG TTT ATC-3′, R: 5′-CAA AAA TAA AAT CTT CTT TTC CGA A-3′; non-methylation F: 5′-AGG AGT TGG GAG TTG TTT ATT-3′, R: 5′-ATT TCA AAA ATA AAA TCT TCT TTT CCA-3′.

### Affymetrix whole-transcript expression array and data analysis

These methods are detailed in the Additional file 1.

### TCGA data

Data regarding the levels of *PTPRD* and *CXCL8* mRNA expression in stomach adenocarcinoma (TCGA, Nature 2014) were extracted from cBioPortal (www.cbioportal.org).

### Statistical analysis

Statistical analyses were performed using SPSS ver.22 (IBM Corp., Armonk, NY, USA). Spearman’s correlation test, Chi-square test, Fisher’s exact test, or unpaired Student’s *t-*test were used, as and when appropriate. Survival analyses were performed using the Kaplan–Meier method and a log-rank test. A *p-*value less than 0.05 was considered statistically significant. All reported *p*-values are two-sided.

## Results

### Loss of PTPRD expression was correlated with advanced cancer stage and metastasis

**Fig. 1:**
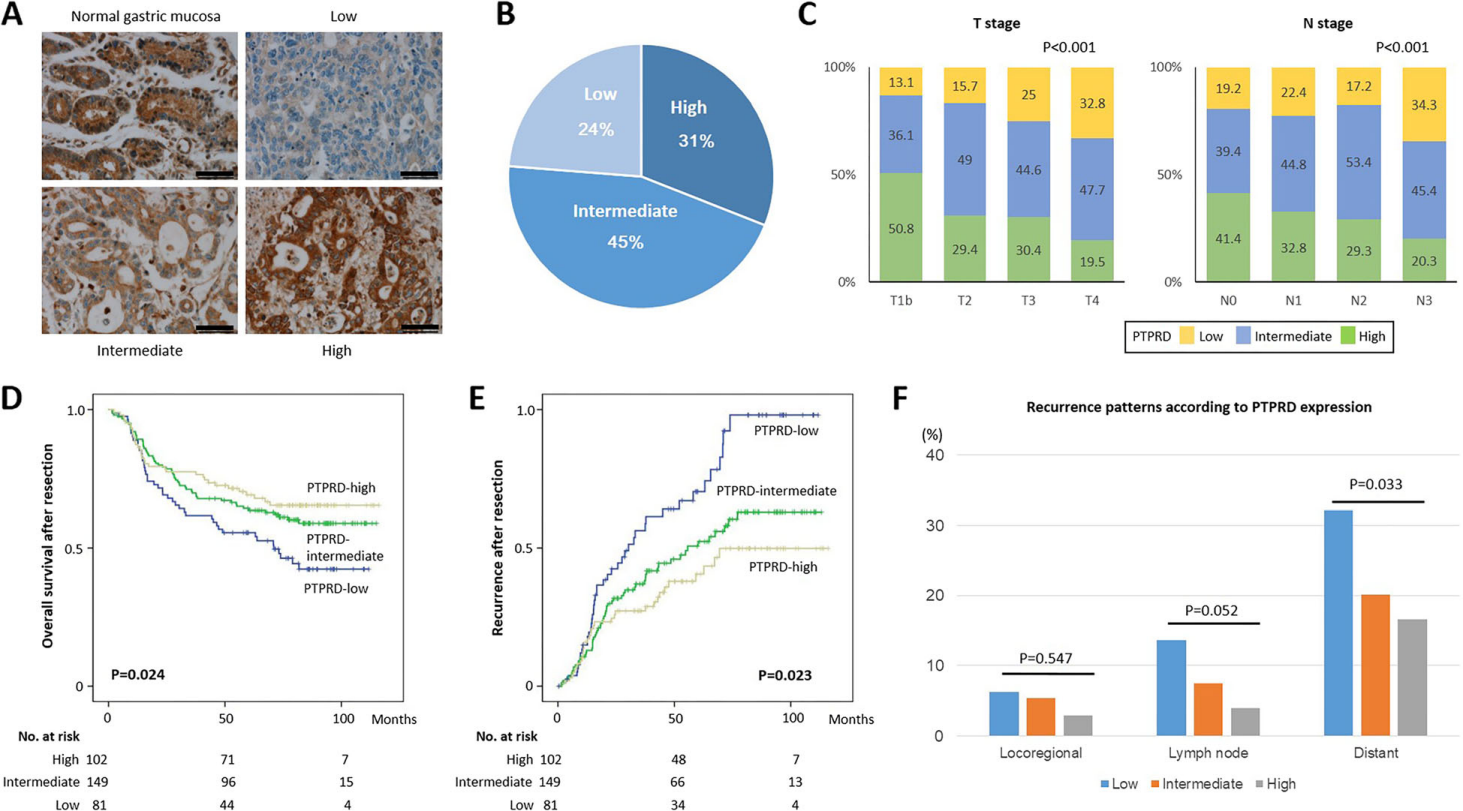
PTPRD expression and its significance in gastric cancer. **a** Representative photomicrographs of immunostaining for PTPRD in normal gastric mucosa and gastric cancer (GC) tissues. **b** The levels of PTPRD expression in GCs. **c** Correlations between PTPRD expression and T or N stages. **d**-**e** Overall survival (**d**) and tumor recurrence (**e**) after surgery were analyzed according to PTPRD expression, using a Kaplan-Meier estimator. The number of patients at risk is indicated. **f** Comparison of recurrence rates according to PTPRD expression at the local region, lymph nodes, and distant sites. Distant sites include other organs, such as the peritoneum, liver, and lungs. Scale bar = 50 μm

To investigate whether PTPRD expression is downregulated in GC, we performed IHC analysis of PTPRD in 20 normal gastric mucosa samples and 332 GC tissues (Fig. 1a). While normal gastric mucosa showed strong cytoplasmic staining for PTPRD, the majority of GCs (230 cases; 69%) showed decreased PTPRD expression (intermediate, 45%; low, 24%; Fig. 1b). Decreased PTPRD expression was associated with diffuse/mixed-type histology, according to the Lauren classification (*p* < 0.001; Additional file 2: Table S1) and was correlated with tumor invasion depth and regional lymph node (LN) metastasis (*p* < 0.001, Fig. 1c). PTPRD expression levels could predict the prognosis of GC patients, with low PTPRD expression levels associated with decreased OS time (*p* = 0.024) and higher recurrence rates after surgery (*p* = 0.023, Fig. 1d-e). Furthermore, we explored recurrence patterns in GC patients after curative surgery and found that low levels of PTPRD expression were associated with a trend toward a higher incidence of cancer recurrence at regional LNs (*p* = 0.052), and significantly predicted distant (including lung, liver, and peritoneum) metastasis (*p* = 0.033, Fig. 1f). Taken together, these results suggest that decreased PTPRD expression is a common event in GC and that the loss of PTPRD expression is associated with aggressive clinical behavior, and in particular, increased rates of metastasis.

### PTPRD negatively regulated CXCL8

**Fig. 2:**
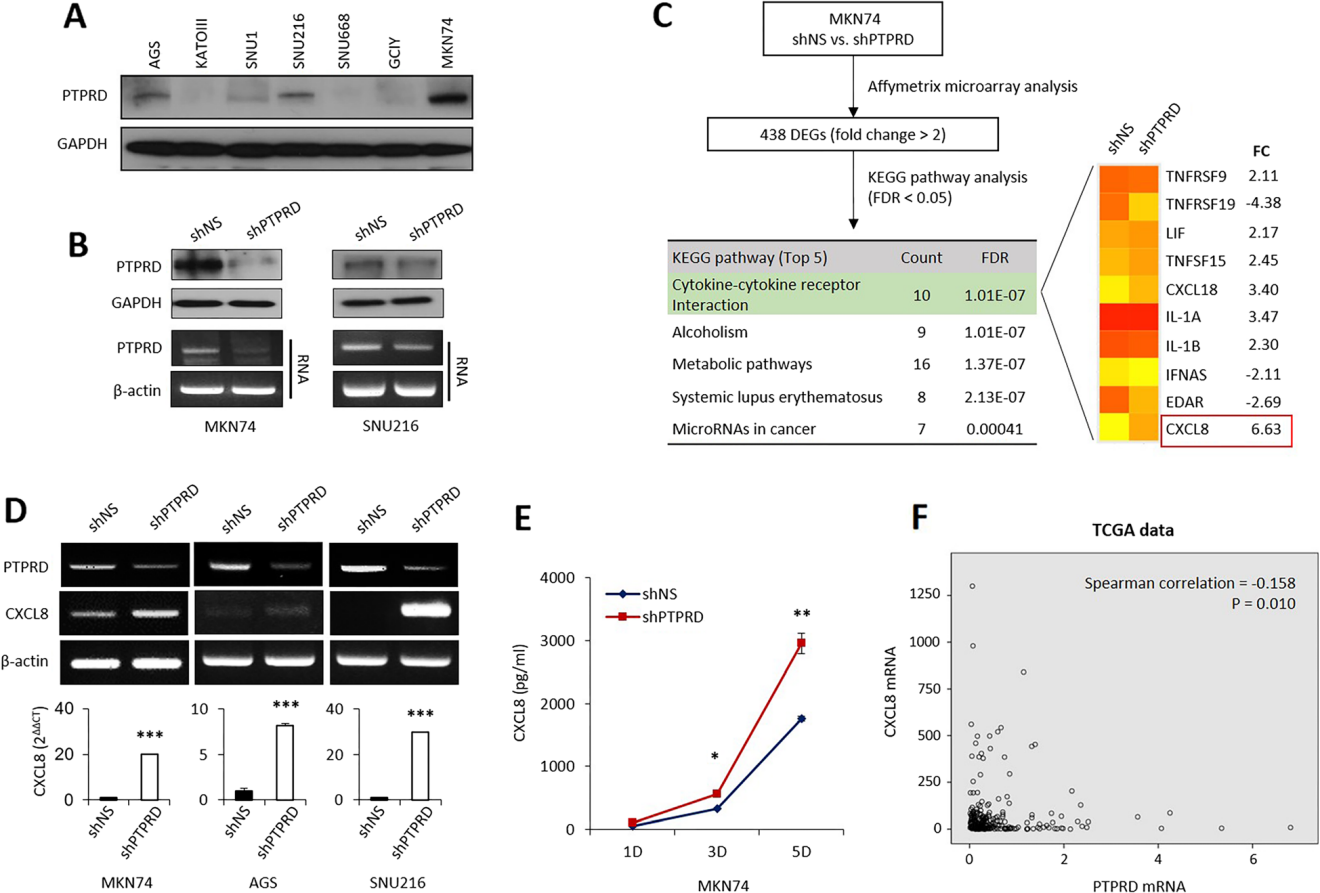
Loss of PTPRD upregulates CXCL8. **a** PTPRD expression in gastric cancer cell lines. **b** PTPRD protein and mRNA expression after lentiviral transduction to silence PTPRD. **c** Summary of the microarray analysis process. KEGG pathway analysis was performed on 438 differentially expressed genes (DEGs). **d** Loss of PTPRD induces the upregulation of CXCL8, as assessed by RT- and qRT-PCR. **e** CXCL8 concentration in the supernatant of cancer cells, with or without PTPRD silencing, as assessed by ELISA. **f** The inverse correlation between *PTPRD* and *CXCL8* mRNA expression in TCGA data. *p < 0.05; **p < 0.01; ***p < 0.001 by unpaired Student’s t*-*test

Immunoblotting analysis of PTPRD expression showed that PTPRD expression was also frequently decreased in GC cell lines (Fig. 2a). After assessing the efficacy of *PTPRD* knockdown by shRNA (Fig. 2b), we determined that stable knockdown of *PTPRD* increased cellular proliferation, as well as cancer cell migration and invasion (Additional file 3: Figure S1), most likely via STAT3, as previously reported [12, 21]. However, we aimed to determine more specific details of the underlying mechanisms with respect to metastasis induced by the loss of PTPRD. Thus, we used an Affymetrix microarray to analyze MKN74 cells transfected with shNS or shPTPRD (Fig. 2c). In total, 438 differentially expressed genes were identified. KEGG pathway analysis showed that cytokine-cytokine receptor interaction was the most significantly altered pathway (false discovery rate [FDR] = 1.01E-07), followed by alcoholism (FDR = 1.01E-07) and metabolic pathways (FDR = 1.37E-07, Additional file 2: Table S2). Ten genes were involved in the cytokine-cytokine receptor interaction pathway and *CXCL8* displayed the greatest fold change (FC: 6.63) in this group. As CXCL8 is a potent pro-angiogenic factor that may also promote cancer growth and metastasis [22], it appeared that the PTPRD-CXCL8 axis may be responsible for cancer metastasis.

Next, to confirm whether PTPRD truly regulates CXCL8, we silenced PTPRD in several GC cell lines using shRNAs and/or siRNAs. PTPRD knockdown led to a marked upregulation of CXCL8 in all cells (Fig. 2d and Additional file 3: Figure S2A). We proceeded to perform quantitative ELISA to measure CXCL8 levels in conditioned media collected from cancer cells treated with shNS or shPTPRD. PTPRD-silenced MKN74 cells were found to secrete significantly more CXCL8 over time than the control cells (Fig. 2e). A similar result was obtained when SNU216 cells were used (Additional file 3: Figure S2B). Finally, using TCGA data (*n* = 265), we identified a significant inverse correlation between *PTPRD* and *CXCL8* mRNA expression in GC samples (*p* = 0.010, Fig. 2f). Taken together, these data strongly support our hypothesis that the loss of PTPRD leads to the upregulation of CXCL8.

### Loss of PTPRD promoted angiogenesis via CXCL8

**Fig. 3:**
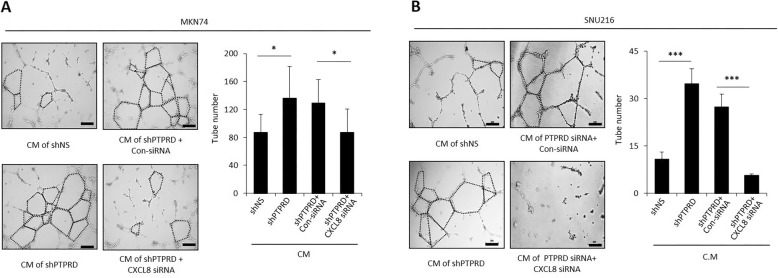
CXCL8 induced by the loss of PTPRD promotes angiogenesis. **a**-**b** In vitro tube formation assays were performed using MKN74 and SNU216 cells. Cancer cells were transfected with shNS or shPTPRD and then treated with control (con) siRNA or CXCL8 siRNA for 48 h. The media were then changed to supplement-free HUVEC media, cells were incubated for 24 h, and conditioned media were collected. HUVECs were incubated in conditioned media for 6–10 h and then tube formation was assessed. These data are representative of three independent experiments. *p < 0.05; ***p < 0.001 by unpaired Student’s t*-*test. Scale bar = 200 μm

To determine whether the loss of PTPRD promotes angiogenesis via CXCL8, we performed an in vitro tube formation assay. The tube-forming ability of HUVECs was greatly impaired in supplement-lacking media, whereas the addition of rhCXCL8 significantly increased tube formation (Additional file 3: Figure S3). This confirmed that CXCL8 has a strong proangiogenic effect. After confirming the efficacy of the siRNA against *CXCL8* (Additional file 3: Figure S4), we collected conditioned media from MKN74 cells transfected with shNS or shPTPRD, and used it to treat HUVECs. Conditioned media from control shRNA-transfected cancer cells did not increase tube formation, whereas conditioned media from shPTPRD-transfected cells significantly increased the tube-forming ability of HUVECs (Fig. 3a). This enhanced tube-forming ability was substantially decreased when CXCL8 was silenced by siCXCL8. Similar results were observed in SNU216 cells (Fig. 3b). Thus, these findings strongly indicate that the loss of PTPRD enhances angiogenesis by upregulating CXCL8.

### CXCL8 expression induced by the loss of PTPRD was mediated by both ERK and STAT3

**Fig. 4:**
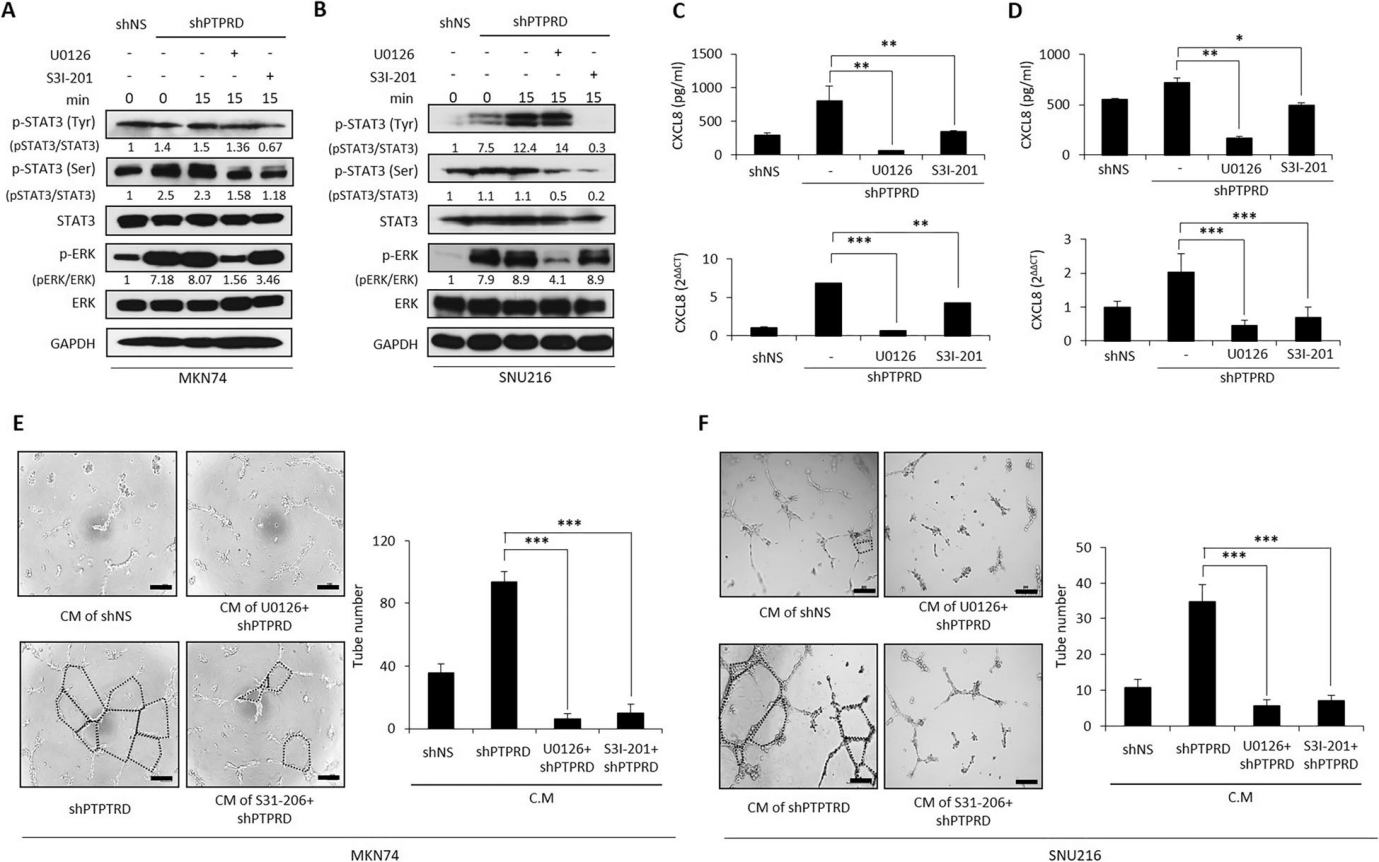
Loss of PTPRD activates ERK and STAT3 signaling. **a-b** Western blots after treatment of MKN74 and SNU216 cells with U0126 (ERK inhibitor) and S3I-201 (STAT3 inhibitor). Cells were collected after pretreatment with U0126 (10 μM) and S3I-201 (100 μM) for 4 h. **c-d** *CXCL8* mRNA and secreted CXCL8 were measured by qRT-PCR and ELISA, respectively. Cancer cells were treated with each inhibitor for 48 h. **e-f** In vitro tube formation assay using MKN74 and SNU216 cells. Lentivirus-transfected cancer cells were treated with control (con) siRNA or CXCL8 siRNA for 48 h and the media were then changed to supplement-free HUVEC media. After 24 h, HUVECs were incubated in these conditioned media for 6–10 h. These data are representative of three independent experiments. *p < 0.05; **p < 0.01; ***p < 0.001 by unpaired Student’s *t*-test. Scale bar = 200 μm

Next, we investigated the mechanism by which CXCL8 was upregulated upon the loss of PTPRD. As previously described, p-STAT3 is a direct substrate of PTPRD [12] and STAT3 regulates CXCL8 [23]. There is also evidence to suggest that ERK regulates CXCL8 [24, 25], although ERK and STAT3 may crosstalk [26]. To determine whether CXCL8 expression induced by the loss of PTPRD is mediated by STAT3 or ERK, we performed immunoblotting for p-STAT3 and p-ERK after stable knockdown of PTPRD. As expected, PTPRD silencing increased the phosphorylation of STAT3 and to an even greater extent, ERK, in both MKN74 (Fig. 4a) and SNU216 cells (Fig. 4b). Consistent with this, the extent of the increase in CXCL8 expression upon loss of PTPRD was substantially reduced by the ERK inhibitor, U0126, and to a greater extent, by the STAT3 inhibitor, S31–201. Both these inhibitors significantly reduced the RNA and protein levels of CXCL8 that were induced by PTPRD knockdown (Fig. 4c-d). Subsequently, we explored the anti-angiogenic effects of U0126 and S31–201 in cancer cells with stable PTPRD knockdown. Both inhibitors significantly, and almost equally, inhibited the tube-formation induced by PTPRD silencing (Fig. 4e-f). Taken together, these results indicate that the loss of PTPRD increases the phosphorylation of ERK and STAT3 and the upregulation of CXCL8 upon loss of PTPRD appears to be mediated by both ERK and STAT3.

### Metformin efficiently suppressed angiogenesis induced by loss of PTPRD

**Fig. 5:**
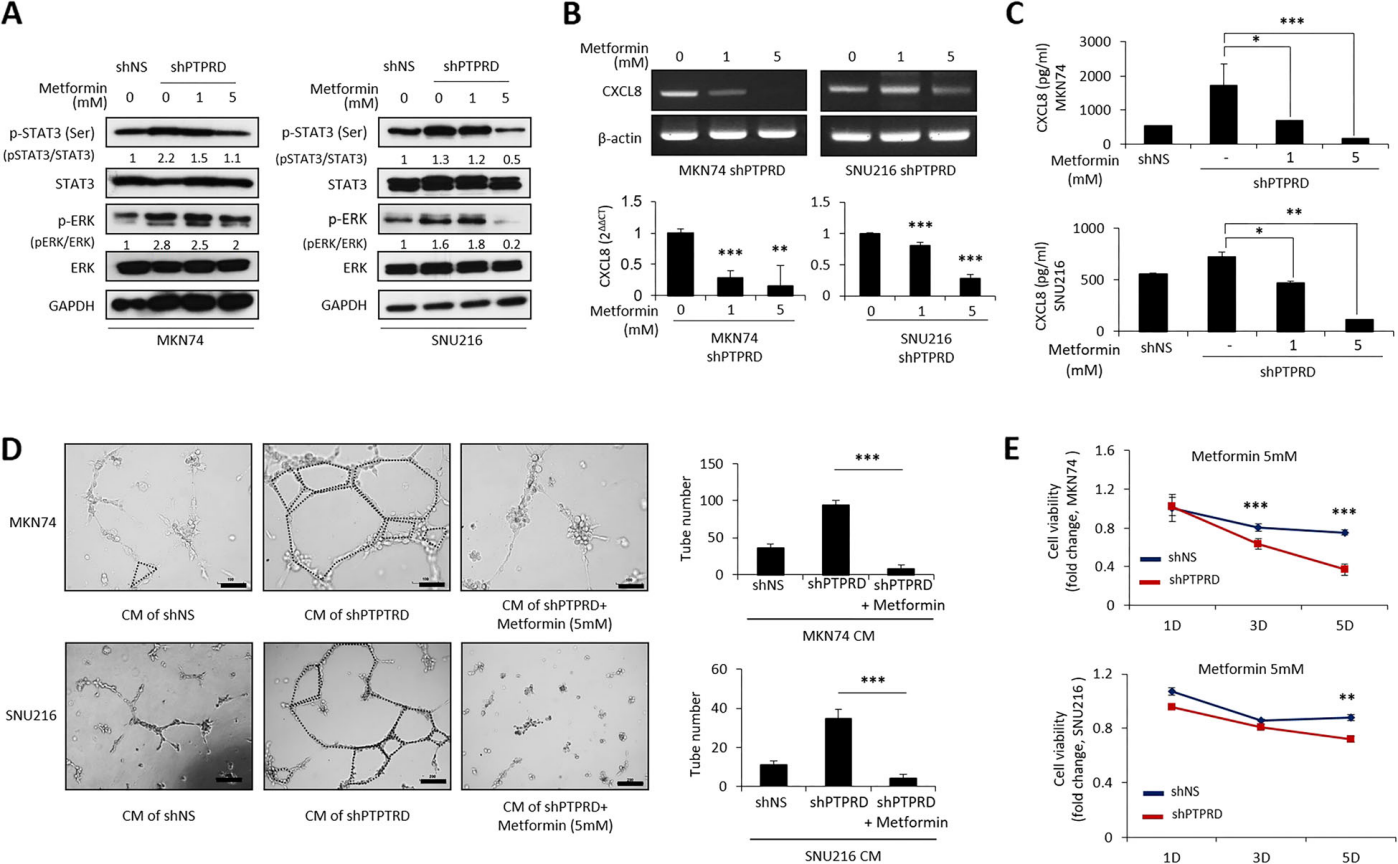
Effect of metformin on PTPRD-inactivated cancer cells. **a** Western blotting after treatment with metformin. Cancer cells were collected 15 min after treatment with metformin. **b** *CXCL8* mRNA expression was assessed by RT−/qRT-PCR after treatment with metformin for 72 h. **c** The amount of secreted CXCL8 was assessed by ELISA after treatment with metformin for 72 h. **d** In vitro tube formation assay after treatment with metformin. Cancer cells were treated with metformin for 72 h and the media were changed to HUVEC media. After 24 h, HUVECs were incubated with these conditioned media for 6–10 h. **e** Cell viability was assessed by MTS assay 1, 3, and 5 days after 5 mM metformin treatment. These data are representative of three independent experiments. *p < 0.05; **p < 0.01; ***p < 0.001 by unpaired Student’s *t-*test. Scale bar = 200 μm

As the loss of PTPRD activated both ERK and STAT3 signaling, a single specific inhibitor would not suffice in patients with PTPRD-silenced cancer. Thus, we performed a literature search for a clinically available drug that inhibits both ERK and STAT3. This led us to metformin, the first line medication for type 2 diabetes, which inhibits the ERK pathway [27] as well as STAT3 signaling [28]. Moreover, a genome-wide association study indicated that single nucleotide polymorphisms in the *PTPRD* gene are strongly associated with type 2 diabetes [29]. Consistent with this, PTPRD silencing leads to type 2 diabetes in an experimental mouse model [30]. Therefore, these findings prompted us to evaluate the therapeutic effect of metformin in PTPRD-reduced cancers. Firstly, we observed that metformin decreased the phosphorylation of both STAT3 and ERK in a dose-dependent manner in PTPRD-silenced GC cells (Fig. 5a). Consequently, metformin decreased CXCL8 expression in a dose-dependent manner, as assessed by qRT-PCR and ELISA (Fig. 5b-c). Additionally, we observed that metformin efficiently inhibited the tube-forming ability of HUVECs induced by PTPRD knockdown (Fig. 5d). Furthermore, metformin reduced cell viability more efficiently when PTPRD was silenced (Fig. 5e). Collectively, these findings strongly indicate that metformin can serve as an effective treatment for patients with GC exhibiting the loss of PTPRD expression.

### Metformin reversed the decrease in PTPRD expression

**Fig. 6:**
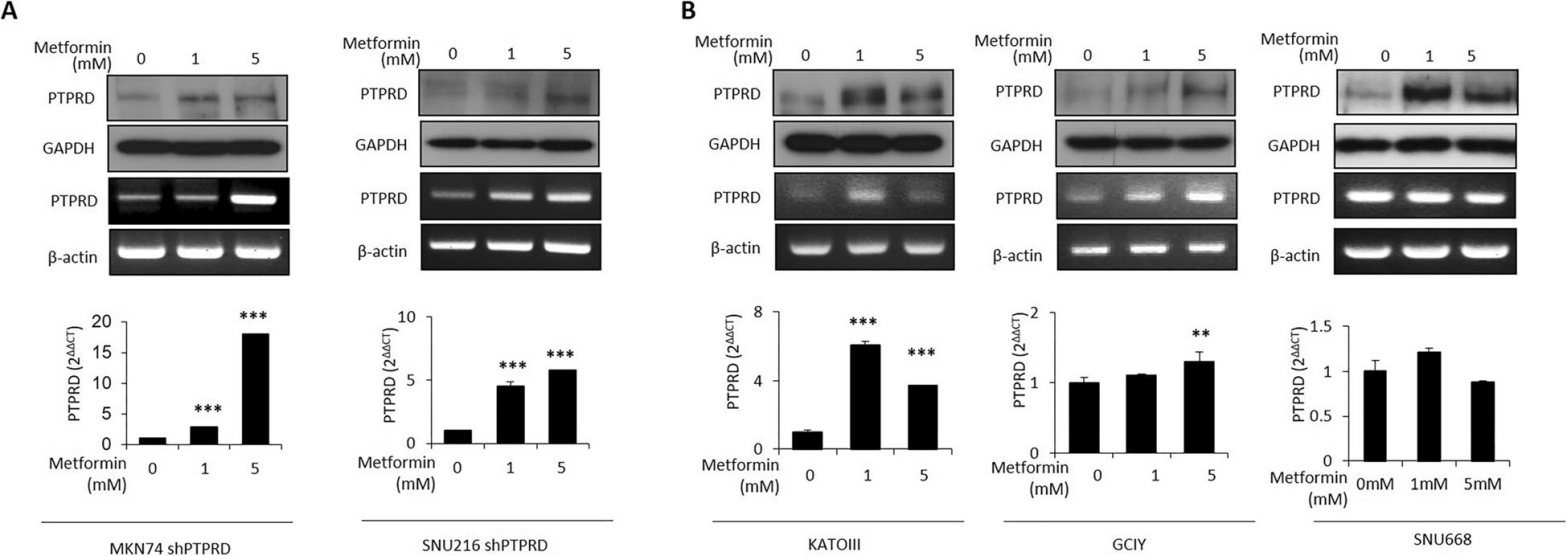
Metformin reverses the reduced PTPRD expression in cancer cells. **a** Protein and mRNA expression levels of PTPRD were assessed by western blotting and RT/qRT-PCR, respectively, upon metformin treatment of MKN74 and SNU216 cells with stable PTPRD knockdown. **b** Protein and mRNA expression levels of PTPRD were assessed by western blotting and RT−/qRT-PCR, respectively, upon metformin treatment of KATOIII, GCIY, and SNU668 cells, in which PTPRD expression is relatively downregulated. These data are representative of three independent experiments. **p < 0.01; ***p < 0.001 by unpaired Student’s *t-*test

As genetic alterations of PTPRD are related to the occurrence of type 2 diabetes [29] and metformin is a medication for this disease, we aimed to determine whether metformin influenced PTPRD expression in cancer cells. Unexpectedly, metformin significantly increased the protein and RNA levels of PTPRD in a dose-dependent manner in MKN74 and SNU216 cells with stable PTPRD knockdown (Fig. 6a), despite the fact that 5 mM metformin treatment significantly reduced cell viability. To further confirm this result, we performed the same experiments in other wild-type GC cell lines with low PTPRD expression, including KATOIII, GCIY, and SNU668. PTPRD promoter methylation was detected in KATOIII and GCIY (Additional file 3: Figure S5). We observed a substantial increase in PTPRD expression upon metformin treatment in all of these cells (Fig. 6b). However, this phenomenon was not observed in wild-type MKN74, SNU216, or AGS cells, in which PTPRD expression was not affected (Additional file 3: Figure S6). Taken together, these results indicate that metformin could increase PTPRD expression, particularly in cells expressing low levels of PTPRD.

## Discussion

Angiogenesis is essential to provide oxygen and nutrients to rapidly growing cancer cells, and it is considered to be one of the most crucial steps during cancer progression [31]. It is now accepted that angiogenesis is a step of paramount importance for the spread and establishment of cancer metastases [32]. Therefore, many research groups have invested considerable effort in inhibiting this critical and rate-limiting step. Unfortunately, these efforts have not yet resulted in significantly tangible improvements to the clinical outcomes of the patients. In 2014, the U.S. Food and Drug Administration (FDA) approved the use of ramucirumab—a monoclonal antibody against vascular endothelial growth factor receptor-2 (VEGFR-2), which was initially used as a monotherapy and subsequently, administered as a combination therapy with paclitaxel—for GC patients. The survival benefits of ramucirumab, however, are only marginal [33]. This marginal impact may stem from the lack of a comprehensive understanding of the different gene-gene and gene-microenvironment interactions promoting angiogenesis in each cancer type. Thus, the identification of a key regulator of angiogenesis in each cancer type may improve outcomes for cancer patients.

The overexpression of CXCL8 in cancer cells has been recognized for many years. Previous studies have identified the critical role of CXCL8 signaling in angiogenesis and metastasis in GC [22, 34]. Furthermore, CXCL8 enhances the resistance of cancer cells to anoikis, thus promoting their metastatic potential [35]. However, the mechanisms underlying CXCL8 overexpression in GC have not been well elucidated. Here, we demonstrated that PTPRD is frequently inactivated in GCs and that loss of PTPRD significantly upregulates CXCL8 via both ERK and STAT3 signaling. This suggests that the PTPRD-ERK/STAT3-CXCL8 axis is the main pathway promoting cancer angiogenesis in PTPRD-inactivated cancers. Furthermore, disrupting the PTPRD-CXCL8 axis may serve as a feasible approach to block angiogenesis and cancer metastasis in a subset of GCs. In this regard, the FDA-approved ramucirumab may not be effective in PTPRD-inactivated cancers, as previously reported [36]. Rather, the use of ramucirumab can induce tissue hypoxia, which may, in turn, result in the induction of more angiogenic factors, such as CXCL8 [37], paradoxically resulting in angiogenesis. In fact, clinical studies have shown that once treated with an anti-angiogenic drug, cancer cells can become more aggressive, with increased local invasion and distant metastases [38, 39].

In the present study, PTPRD expression was diminished in 55% of the human GC tissues, as assessed by IHC. This frequency is similar to that observed in a previous study, which reported that 50.87% of GC samples had reduced PTPRD expression [40]. PTPRD expression is commonly downregulated by genetic or epigenetic alterations in various cancer types, including glioblastoma and head and neck cancer [12]. As mutations and deep deletions of PTPRD are found in approximately 15% of GCs, based on TCGA data [6], epigenetic alterations, such as promoter methylation may be responsible for the remaining cases of PTPRD downregulation, as we found in some GC cell lines. The exact prevalence of PTPRD promoter methylation in association with the loss of PTPRD expression needs to be investigated in future studies.

As the potential relevance of metformin to cancer risk was first suggested in 2005 [18], the anti-neoplastic action of metformin has been intensely investigated [41]. However, the exact anticancer mechanism remains unknown, because metformin has multifaceted functions. Given the relationship between PTPRD and diabetes [29, 30], we decided to treat PTPRD-inactivated cancer cells with metformin. Metformin treatment inhibited both ERK and STAT3 signaling, significantly reduced CXCL8 expression, and consequently, decreased tube-forming ability induced by the loss of PTPRD. These findings indicate that metformin may display significant anti-angiogenic effects in a subset of GCs. Consistent with this, a previous study revealed that metformin inhibits the secretion of CXCL8 in human thyroid cells [42]. We also found that metformin selectively decreased the viability of cells with stable PTPRD knockdown. As PTPRD-inactivated cancers are more dependent on ERK and STAT3 signaling, it seems plausible that PTPRD-inactivated cancers are more vulnerable to metformin due to its inhibition of both critical signaling pathways. Therefore, the use of metformin may be an effective strategy in the treatment of PTPRD-inactivated cancers, as it not only reduces cellular proliferation, but also decreases angiogenesis in those cancers. As metformin is already in use and its stability has been verified, clinical studies using metformin to treat PTPRD-inactivated GCs appear promising.

More intriguingly, we discovered that metformin could reverse the decreased PTPRD expression in cancer cells. Initially, we identified this effect in cancer cells with stable PTPRD knockdown and we then confirmed it in wild-type GC cell lines. We found that metformin increased PTPRD expression in cancer cells, especially when PTPRD expression was downregulated. Therefore, metformin appears to exert its effect on cancer cells not only by inhibiting ERK and STAT3 signaling, but also by reversing the downregulated PTPRD expression. Assuming PTPRD inactivation is one of the driving events in cancer initiation, activating PTPRD by metformin may hinder cancer growth in many different ways. A previous study also supports this notion, as it demonstrated that the *PTPRD* rs17584499 C/T polymorphism is associated with improved postprandial glucose and glycated hemoglobin (HbA1c) levels in Chinese type 2 diabetes patients treated with metformin monotherapy [43]. Considering the association between PTPRD polymorphisms and the incidence of type 2 diabetes, PTPRD may be critical for cellular metabolism and re-activating the expression of this gene appears to be important for treating diabetes as well as PTPRD-inactivated cancers. This interesting medication-gene interaction and its underlying regulatory mechanisms warrants thorough investigation in future studies.

Our study has several limitations. Firstly, we did not investigate metastasis induced by the loss of PTPRD in an in vivo model, mainly because of the lack of a genetic mouse model of GC and technical difficulties in establishing an orthotopic GC model. Secondly, although we showed that the loss of PTPRD significantly increased p-ERK, we did not investigate the direct mechanism whereby PTPRD regulates ERK. Finally, although we demonstrated the effect of metformin on PTPRD expression, we did not specify its mechanism of action, as the upstream regulator of PTPRD is currently unknown.

## Conclusions

In summary, we demonstrated that PTPRD is frequently inactivated in GC and the loss of PTPRD induces CXCL8, thus promoting angiogenesis and consequently, metastasis, via both ERK and STAT3 signaling. Therefore, inhibiting the PTPRD-CXCL8 axis may serve as a promising option for treating GC metastasis. Additionally, we showed that metformin may be an efficient strategy for the treatment of PTPRD-inactivated GCs, as it efficiently inhibited cancer angiogenesis and growth and reversed the decrease in PTPRD expression. Identification of the exact regulatory mechanism underlying the effects of metformin on PTPRD expression would provide further support for its use in cancer therapy.

## Supplementary information

**Additional file 1.** Supplementary Methods

**Additional file 2: Table S1.** The clinicopathological characteristics of 332 gastric cancer patients according to the PTPRD expression statuses. **Table S2** Significantly altered pathways by PTPRD-knockdown in MKN74 gastric cancer cell line.

**Additional file 3: Figure S1.** PTPRD silencing promotes cellular proliferation and migragion/invasion. **Figure S2.** Loss of PTPRD induce upregulation of CXCL8. **Figure S3.** HUVECs were cultured in supplement-lacking media with or without 1 ng/ml human recombinant CXCL8 (rhCXCL8). **Figure S4.** (A) MKN74 cells were treated with con-siRNA or CXCL8 siRNA for 48 h. **Figure S5.** PTPRD promoter methylation was assessed in various gastric cancer cell lines using methylation-specific PCR assay. **Figure S6.** The protein and mRNA expression of PTPRD were assessed upon treatment with metformin by western blot and RT/qRT-PCR using KATOIII, GCIY, and SNU668 whose PTPRD expression was relatively spared.

## Data Availability

The datasets used and analyzed in the current study are available from the corresponding author on reasonable request.

## Abbreviations

ERK: Extracellular signal regulated kinase
FDA: Food and drug administration
GC: Gastric cancer
GWAS: Genome wide association study
HUVEC: Human umbilical vein endothelial cell
IHC: Immunohistochemistry
PTP: Protein tyrosine phosphatase
PTPRD: Protein tyrosine phosphatase receptor delta
STAT3: Signal transducer and activator of transcription 3
TCGA: The cancer genome atlas
VEGFR: Vascular endothelial growth factor receptor
