# Supplementary material for: PTPRD-inactivation-induced CXCL8 promotes angiogenesis and metastasis in gastric cancer and is inhibited by metformin
Source: J Exp Clin Cancer Res. 2019 Dec 5;38:484. doi: 10.1186/s13046-019-1469-4 (PMC6896474; doi:10.1186/s13046-019-1469-4)
Supplement: Supplementary file 1 — Additional file 1. Supplementary Methods [file 13046_2019_1469_MOESM1_ESM.docx]

**Supplementary Methods**

**Affymetrix whole transcript expression array method**

The Affymetrix Whole transcript Expression array process was executed according to the manufacturer's protocol (GeneChip Whole Transcript PLUS reagent Kit). cDNA was synthesized using the GeneChip WT (Whole Transcript) Amplification kit as described by the manufacturer. The sense cDNA was then fragmented and biotin-labeled with TdT (terminal deoxynucleotidyl transferase) using the GeneChip WT Terminal labeling kit. Approximately 5.5 μg of labeled DNA target was hybridized to the Affymetrix GeneChip Human 2.0 ST Array at 45°C for 16hour. Hybridized arrays were washed and stained on a GeneChip Fluidics Station 450 and scanned on a GCS3000 Scanner (Affymetrix). Signal values were computed using the Affymetrix® GeneChip™ Command Console software.

**Raw data preparation and statistical analysis**

Raw data were extracted automatically in Affymetrix data extraction protocol using the software provided by Affymetrix GeneChip® Command Console® Software (AGCC). After importing CEL files, the data were summarized and normalized with robust multi-average (RMA) method implemented in Affymetrix® Expression Console™ Software (EC). We exported the result with gene level RMA analysis and perfomed the differentially expressed gene (DEG) analysis. The comparative analysis between test sample and control sample was carried out using fold change. Gene-Enrichment and Functional Annotation analysis for significant probe list was performed using gene ontology (<http://geneontology.org/>). All Statistical test and visualization of differentially expressed genes was conducted using R statistical language v. 3.1.2. ([www.r-project.org](http://www.r-project.org)).

**The primer sequence information used for RT-PCR and qRT-PCR.**

| Gene |  | Sequence (5’-3’) | Size (bp) | Tm (°C) | cycle |
| --- | --- | --- | --- | --- | --- |
| PTPRD | F | CTG GTT GCT TCA TCG TCA TAG A | 101 | 60 | RT : 34~38  qRT : 35 |
|  | R | GTT CCT CTG GGC TCT CAT TAA A |  |  |  |
| CXCL8 | F | CCA CCG GAG CAC TCC ATA AG | 97 | 60 |  |
|  | R | GAT GGT TCC TTC CGG TGG TT |  |  |  |
| ­β-actin | F | GGG TCA GAA GGA TTC CTA TG | 238 | 60 |  |
|  | R | GGT CTC AAA CAT GAT CTG GG |  |  |  |
