# Supplementary material for: PTPRD-inactivation-induced CXCL8 promotes angiogenesis and metastasis in gastric cancer and is inhibited by metformin
Source: J Exp Clin Cancer Res. 2019 Dec 5;38:484. doi: 10.1186/s13046-019-1469-4 (PMC6896474; doi:10.1186/s13046-019-1469-4)
Supplement: Supplementary file 2 — Additional file 2: Table S1. The clinicopathological characteristics of 332 gastric cancer patients according to the PTPRD expression statuses. Table S2 Significantly altered pathways by PTPRD-knockdown in MKN74 gastric cancer cell line. [file 13046_2019_1469_MOESM2_ESM.docx]

| Variables |  | PTPRD-high (n=102) | |  | PTPRD-intermediate (n=149) | |  | PTPRD-low (n=81) | |  | P value |
| --- | --- | --- | --- | --- | --- | --- | --- | --- | --- | --- | --- |
|  | n | n | % |  | n | % |  | n | % |  |  |
| Age |  |  |  |  |  |  |  |  |  |  | 0.314 |
| ≤ 60 | 154 | 41 | 26.6 |  | 74 | 48.1 |  | 39 | 25.3 |  |  |
| >60 | 178 | 61 | 34.3 |  | 75 | 42.1 |  | 42 | 23.6 |  |  |
| Gender |  |  |  |  |  |  |  |  |  |  | 0.378 |
| Male | 229 | 71 | 31.0 |  | 107 | 46.7 |  | 51 | 22.3 |  |  |
| Female | 103 | 31 | 30.1 |  | 42 | 40.8 |  | 30 | 29.1 |  |  |
| Tumor size |  |  |  |  |  |  |  |  |  |  | 0.059 |
| ≤ 5cm | 157 | 52 | 33.1 |  | 76 | 48.4 |  | 29 | 18.5 |  |  |
| > 5cm | 175 | 50 | 28.6 |  | 73 | 41.7 |  | 52 | 29.7 |  |  |
| Tumor location |  |  |  |  |  |  |  |  |  |  | 0.770 |
| upper | 64 | 16 | 25.0 |  | 31 | 48.4 |  | 17 | 26.6 |  |  |
| mid | 89 | 26 | 29.2 |  | 40 | 44.9 |  | 23 | 25.8 |  |  |
| lower | 179 | 30 | 16.8 |  | 78 | 43.6 |  | 41 | 22.9 |  |  |
| Histology |  |  |  |  |  |  |  |  |  |  | <0.001* |
| WD | 37 | 18 | 48.6 |  | 16 | 43.2 |  | 3 | 8.1 |  |  |
| MD | 101 | 31 | 30.7 |  | 53 | 52.5 |  | 17 | 16.8 |  |  |
| PD | 104 | 37 | 35.6 |  | 40 | 38.5 |  | 27 | 26.0 |  |  |
| Signet ring cell | 75 | 13 | 17.3 |  | 30 | 40.0 |  | 32 | 42.7 |  |  |
| Mucinous | 15 | 3 | 20.0 |  | 10 | 66.7 |  | 2 | 13.3 |  |  |
| Lauren classification | |  |  |  |  |  |  |  |  |  | <0.001* |
| Intestinal | 202 | 75 | 37.1 |  | 96 | 47.5 |  | 31 | 15.3 |  |  |
| diffuse | 105 | 20 | 19.0 |  | 45 | 42.9 |  | 40 | 38.1 |  |  |
| mixed | 25 | 7 | 28.0 |  | 8 | 32.0 |  | 10 | 40.0 |  |  |
| T stage |  |  |  |  |  |  |  |  |  |  | <0.001† |
| T1b | 61 | 31 | 50.8 |  | 22 | 36.1 |  | 8 | 13.1 |  |  |
| T2 | 51 | 15 | 29.4 |  | 25 | 49.0 |  | 8 | 15.7 |  |  |
| T3 | 92 | 28 | 30.4 |  | 41 | 44.6 |  | 23 | 25.0 |  |  |
| T4 | 128 | 25 | 19.5 |  | 61 | 47.7 |  | 42 | 32.8 |  |  |
| N stage |  |  |  |  |  |  |  |  |  |  | <0.001† |
| N0 | 99 | 41 | 41.4 |  | 39 | 39.4 |  | 19 | 19.2 |  |  |
| N1 | 67 | 22 | 32.8 |  | 30 | 44.8 |  | 15 | 22.4 |  |  |
| N2 | 58 | 17 | 29.3 |  | 31 | 53.4 |  | 10 | 17.2 |  |  |
| N3a | 64 | 14 | 21.9 |  | 30 | 46.9 |  | 20 | 31.3 |  |  |
| N3b | 44 | 8 | 18.2 |  | 19 | 43.2 |  | 17 | 38.6 |  |  |
| Lymphovascular invasion | |  |  |  |  |  |  |  |  |  | 0.283 |
| Absent | 160 | 45 | 28.1 |  | 79 | 49.4 |  | 36 | 22.5 |  |  |
| Present | 172 | 57 | 33.1 |  | 70 | 40.7 |  | 45 | 26.2 |  |  |
| Neural/perineural invasion | |  |  |  |  |  |  |  |  |  | 0.002* |
| Absent | 222 | 81 | 36.5 |  | 96 | 43.2 |  | 45 | 20.3 |  |  |
| Present | 110 | 21 | 19.1 |  | 53 | 48.2 |  | 36 | 32.7 |  |  |
| EBV infection |  |  |  |  |  |  |  |  |  |  | 0.568 |
| Absent | 301 | 90 | 29.9 |  | 136 | 45.2 |  | 75 | 24.9 |  |  |
| Present | 31 | 12 | 38.7 |  | 13 | 41.9 |  | 6 | 19.4 |  |  |

**Additional file 2: Table S1.** The clinicopathological characteristics of 332 gastric cancer patients according to the PTPRD expression statuses.

WD, well differentiated; MD, moderately differentiated, PD, poorly differentiated

†Chi-square test by two-sided linear-by-linear association

*Significant at the level of P < 0.05

**Table S2.** Significantly altered pathways by PTPRD-knockdown in MKN74 gastric cancer cell line.

| KEGG pathway | Counts | Genes | FDR |
| --- | --- | --- | --- |
| Cytokine-cytokine receptor  interaction | 10 | TNFRSF9, IL18, TNFRSF19, EDAR, IL1A, IL1B, LIF, CXCL8, IFNA5, TNFSF15 | 1.0126E-07 |
| Alcoholism | 9 | HIST2H2AB, HIST1H2BI, HIST1H2AI, HIST1H2BB, HIST1H4D, HIST1H3F, HIST1H2AJ, HIST1H2AK, SHC3 | 1.0126E-07 |
| Metabolic pathways | 16 | DEGS1, GCLM, PTGS2, ACSL5, GANC, CES1, CYP4F3, CYP24A1, POLR3G, GCNT4, NT5E, GCLC, AKR1B10, CYP3A7, TUSC3, GDA | 1.3749E-07 |
| Systemic lupus erythematosus | 8 | HIST2H2AB, HIST1H2BI, HIST1H2AI, HIST1H2BB, HIST1H4D, HIST1H3F, HIST1H2AJ, HIST1H2AK | 2.1346E-07 |
| MicroRNAs in cancer | 7 | PTGS2, MIR29B2, MIR21, CDC25B, CYP24A1, HMOX1, MIR29A | 0.00041656 |
| Toll-like receptor signaling pathway | 5 | IL1B, CXCL8, SPP1, TLR6, IFNA5 | 0.00094641 |
| Cytosolic DNA-sensing pathway | 4 | IL18, IL1B, POLR3G, IFNA5 | 0.00424418 |
| Influenza A | 5 | IL18, IL1A, IL1B, CXCL8, IFNA5 | 0.004487 |
| Tuberculosis | 5 | IL18, IL1A, IL1B, TLR6, IFNA5 | 0.004487 |
| Salmonella infection | 4 | IL18, IL1A, IL1B, CXCL8 | 0.00643461 |
| Hematopoietic cell lineage | 4 | IL1A, IL1B, MME, ITGA2 | 0.00643461 |
| Rheumatoid arthritis | 4 | IL18, IL1A, IL1B, CXCL8 | 0.0066963 |
| Chagas disease  (American trypanosomiasis) | 4 | IL1B, CXCL8, TLR6, SERPINE1 | 0.00900461 |
| Amoebiasis | 4 | SERPINB2, IL1B, FN1, CXCL8 | 0.00954136 |
| Prion diseases | 3 | IL1A, IL1B, EGR1 | 0.01769908 |
| Hippo signaling pathway | 4 | AJUBA, AREG, SERPINE1, AMOT | 0.02190587 |
| Malaria | 3 | IL18, IL1B, CXCL8 | 0.02790912 |
| Legionellosis | 3 | IL18, IL1B, CXCL8 | 0.03277134 |
| NOD-like receptor signaling pathway | 3 | IL18, IL1B, CXCL8 | 0.03320833 |
| Pathways in cancer | 5 | PTGS2, FN1, CXCL8, ITGA2, DAPK1 | 0.03585892 |
| Proteoglycans in cancer | 4 | MIR21, FN1, ITGA2, HBEGF | 0.03585892 |
| Focal adhesion | 4 | FN1, SPP1, ITGA2, SHC3 | 0.03585892 |
| Inflammatory bowel disease (IBD) | 3 | IL18, IL1A, IL1B | 0.03719106 |
| Leishmaniasis | 3 | PTGS2, IL1A, IL1B | 0.04229095 |
| Pertussis | 3 | IL1A, IL1B, CXCL8 | 0.04229095 |
| Chemical carcinogenesis | 3 | PTGS2, AKR1C2, CYP3A7 | 0.04806577 |
| Small cell lung cancer | 3 | PTGS2, FN1, ITGA2 | 0.04813684 |
| ErbB signaling pathway | 3 | AREG, HBEGF, SHC3 | 0.04813684 |
| ECM-receptor interaction | 3 | FN1, SPP1, ITGA2 | 0.04813684 |

KEGG, Kyoto Encyclopedia of Genes and Genomes; FDR, false discovery rate.

Note: Pathways were clustered after gene expression analysis. Total 29 pathways changed significantly (FDR < 0.05).
