# Supplementary material for: PTPRD-inactivation-induced CXCL8 promotes angiogenesis and metastasis in gastric cancer and is inhibited by metformin
Source: J Exp Clin Cancer Res. 2019 Dec 5;38:484. doi: 10.1186/s13046-019-1469-4 (PMC6896474; doi:10.1186/s13046-019-1469-4)
Supplement: Supplementary file 3 — Additional file 3: Figure S1. PTPRD silencing promotes cellular proliferation and migragion/invasion. Figure S2. Loss of PTPRD induce upregulation of CXCL8. Figure S3. HUVECs were cultured in supplement-lacking media with or without 1 ng/ml human recombinant CXCL8 (rhCXCL8). Figure S4. (A) MKN74 cells were treated with con-siRNA or CXCL8 siRNA for 48 h. Figure S5. PTPRD promoter methylation was assessed in various gastric cancer cell lines using methylation-specific PCR assay. Figure S6. The protein and mRNA expression of PTPRD were assessed upon treatment with metformin by western blot and RT/qRT-PCR using KATOIII, GCIY, and SNU668 whose PTPRD expression was relatively spared. [file 13046_2019_1469_MOESM3_ESM.docx]

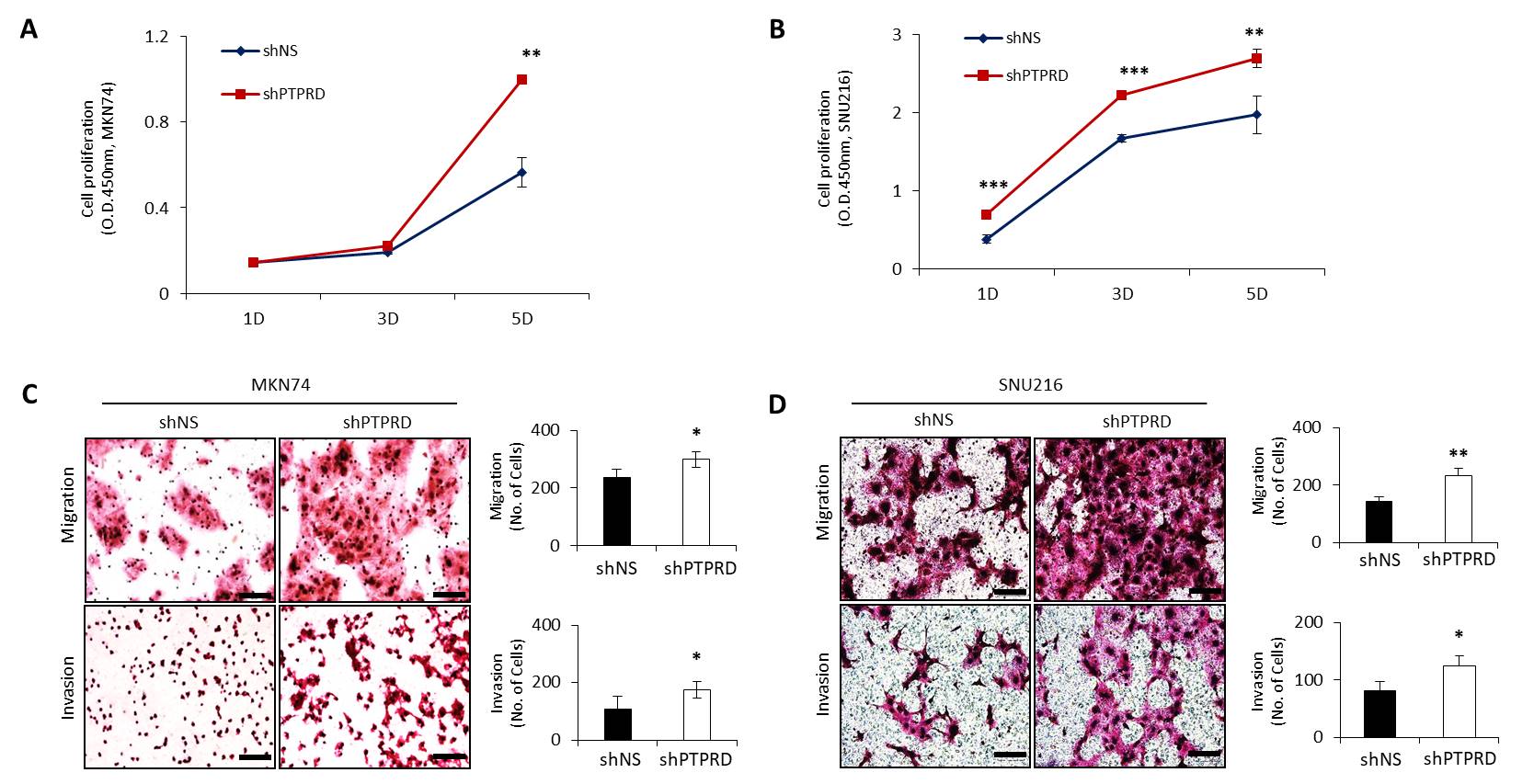


Additional file 3: Figure S1. PTPRD silencing promotes cellular proliferation and migragion/invasion. Cell viability was analyzed using MTS assay in MKN74 and SNU216 cells transfected with shNS and shPTPRD. (C-D). Transwell migration and invasion assays were done using MKN74 and SNU216 cells transfected with shNS and shPTPRD. These data were representative of three independent experiments. **P* < 0.05; ***P* < 0.01; ****P* < 0.001 by unpaired Student’s *t-*test. Bar = 100μm.


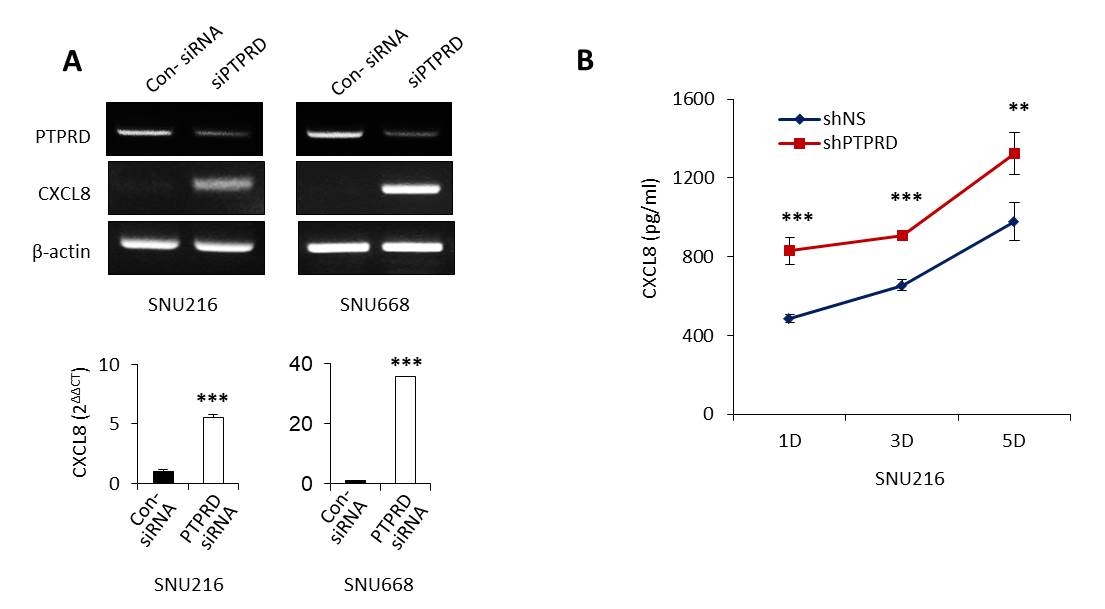


Figure S2. Loss of PTPRD induce upregulation of CXCL8. (A) CXCL8 mRNA expression was analyzed upon PTPRD silencing using siRNA against PTPRD by RT- and qRT-PCR. (B) The amount of CXCL8 in the supernatant of cancer cells with or without PTPRD silencing in SNU216 cells, assessed by ELISA. These data were representative of three independent experiments. ***P* < 0.01; ****P* < 0.001 by unpaired Student’s *t-*test.


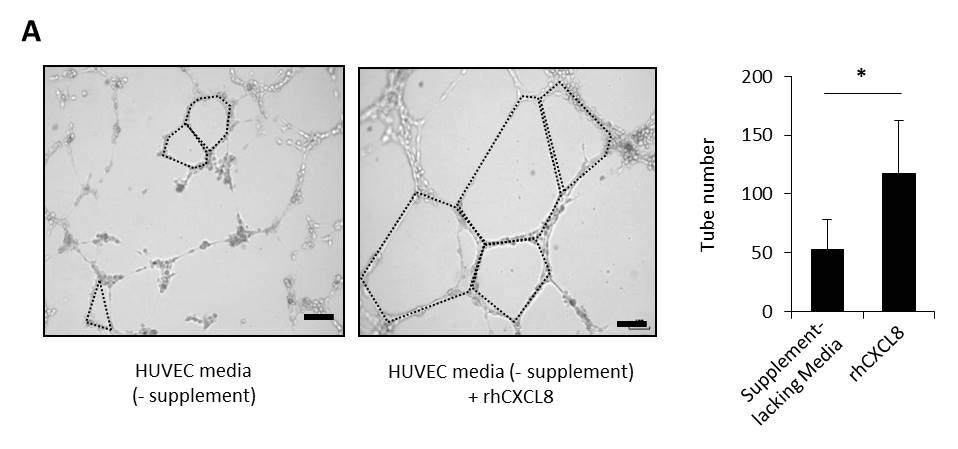


**Figure S3.** HUVECs were cultured in supplement-lacking media with or without 1 ng/ml human recombinant CXCL8 (rhCXCL8). After 6-10 h, tube formations were counted. These data are representative of three independent experiments. **P* < 0.05 by unpaired Student’s *t-*test. Bars = 200 μm.


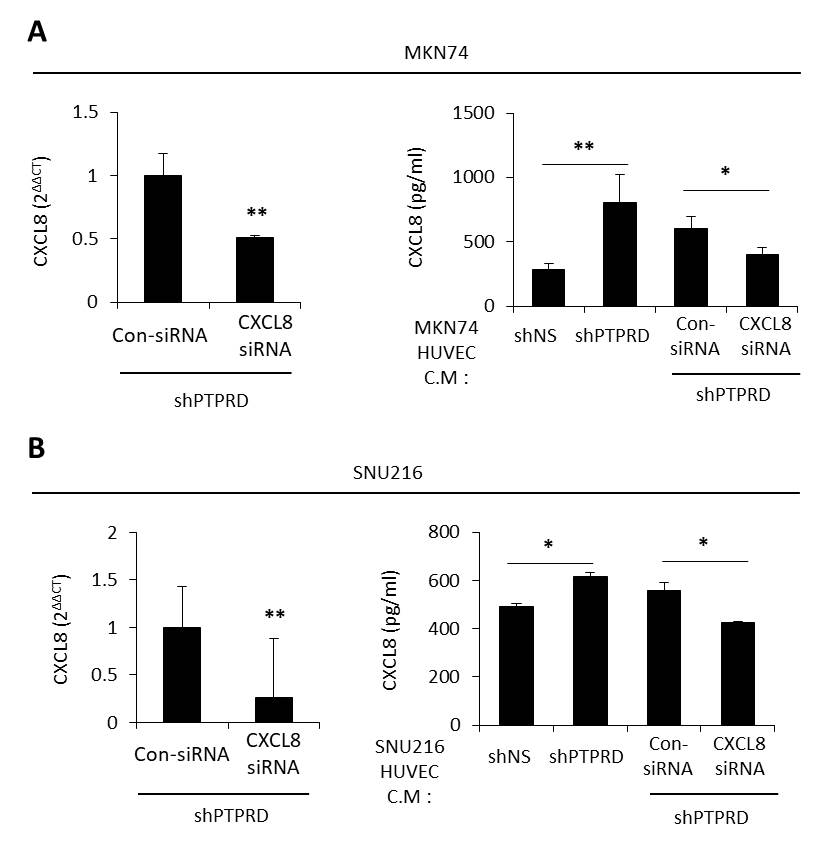


Figure S4. (A) MKN74 cells were treated with con-siRNA or CXCL8 siRNA for 48 h. After transfection, the media was changed to supplement-lacking HUVEC media, and cells were incubated in this media for 24 h. Then, cells or conditioned media was collected, and then CXCL8 mRNA and secreted CXCL8 were assessed by qRT-PCR or ELISA. (B) Same experiments were performed using SNU216 cells. These data are representative of three independent experiments. **P* < 0.05; ***P* < 0.01 by unpaired Student’s *t-*test.


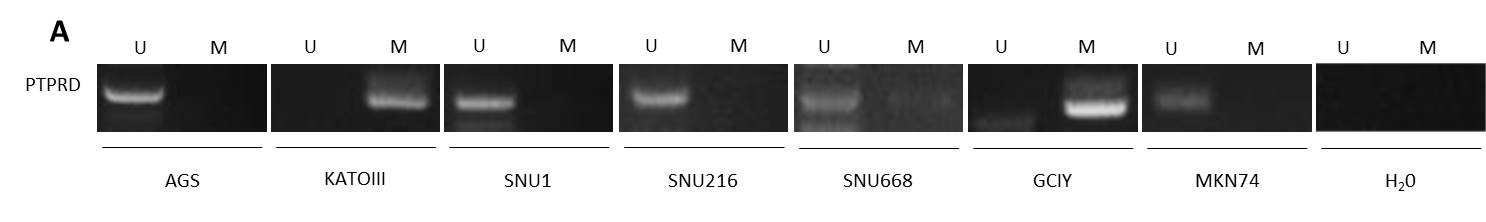


Figure S5. PTPRD promoter methylation was assessed in various gastric cancer cell lines using methylation-specific PCR assay. U denotes the presence of unmethylated alleles, while M denotes the presence of methylated alleles. H_2_O was used as a negative control.

**
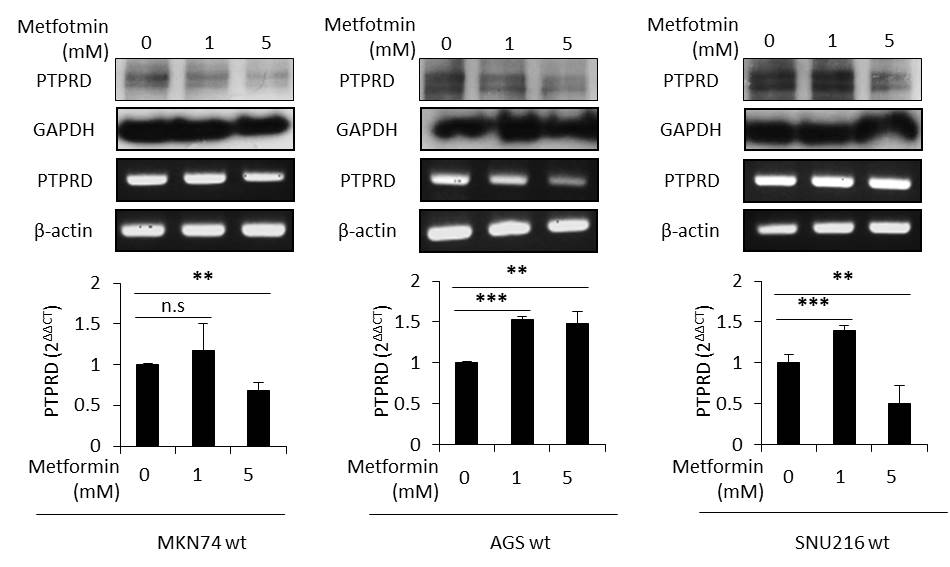
**

Figure S6. The protein and mRNA expression of PTPRD were assessed upon treatment with metformin by western blot and RT/qRT-PCR using KATOIII, GCIY, and SNU668 whose PTPRD expression was relatively spared. These data are representative of three independent experiments. ***P* < 0.01; ****P* < 0.001 by unpaired Student’s *t-*test.
